# The Effects of the Cultivar and Environment on the Phenolic Contents of Hazelnut Kernels

**DOI:** 10.3390/plants11223051

**Published:** 2022-11-11

**Authors:** Anita Solar, Aljaz Medic, Ana Slatnar, Maja Mikulic-Petkovsek, Roberto Botta, Mercè Rovira, Jean-Paul Sarraquigne, Ana Paula Silva, Robert Veberic, Franci Stampar, Metka Hudina, Loretta Bacchetta

**Affiliations:** 1 Department of Agronomy, Biotechnical Faculty, University of Ljubljana, SI 1000 Ljubljana, Slovenia; 2 DISAFA—Dipartimento di Scienze Agrarie, Forestali e Alimentari, Universita’ degli Studi di Torino (UNITO), Grugliasco, 10095 Torino, Italy; 3 Institut de Recerca i Tecnologia Agroalimentàries (IRTA), 08140 Caldes de Montbui, Spain; 4 Association Nationale des Producteurs de Noisette (ANPN), 47290 Cancon, France; 5 Centre for the Research and Technology of Agro-Environmental and Biological Sciences (CITAB), Universidade de Trás-os-Montes e Alto Douro, 5000-801 Vila Real, Portugal; 6 Ente per le Nuove Tecnologie, l’Energia e l’Ambiente (ENEA), 00196 Roma, Italy

**Keywords:** phenolic compounds, *Corylus avellana* L., quality, identification, quantification, HPLC, mass spectrometry

## Abstract

Different climatic conditions are known to affect the synthesis of primary and secondary metabolites. Therefore, the phenolic contents in new growing areas could affect the quality and flavor of hazelnuts. The aim of this study was to determine the variability of the phenolic contents of the kernels in different commercial hazelnut cultivars depending on their growing area. Five cultivars (‘Tonda Gentile delle Langhe’, ‘Merveille de Bollwiller’, ‘Pauetet’, ‘Tonda di Giffoni’, and ‘Barcelona’ (syn. ‘Fertile de Coutard’)) grown in different European collection orchards were included in the study. High-performance liquid chromatography coupled with mass spectrometry was used to identify and quantify the phenolic compounds. Thirteen phenols were identified in the hazelnut kernels, including 7 flavanols, 2 hydroxybenzoic acids, 3 flavonols, and one dihydrochalcone. Catechin and procyanidin dimers were the main phenolic compounds found in the hazelnut kernels. The highest contents of catechin and total flavanols were determined in cultivars cultivated in Spain and northern Italy, and the lowest in Slovenia and France. Flavanols were the major phenolic groups independent of the place of cultivation, as they accounted for more than 50% of all phenolic compounds identified. The flavanols were followed by hydroxybenzoic acids, flavonols, and dihydrochalcones. Higher contents of flavanols and flavonols were found in kernels from areas characterized by higher natural irradiation, which stimulates their accumulation. The contents of hydroxybenzoic acids correlated with altitude, which stimulated phenolic acid synthesis. A negative correlation was observed between the dihydrochalcone content and annual rainfall, probably due to hydric stress.

## 1. Introduction

The hazelnut (*Corylus avellana* L.) is one of the most important nut crops worldwide and ranks third in the global nut market. The global cultivated area is 1,015,216 ha and the production rate is over 1,072,308 tons per year [1]. In terms of altitude, the hazelnut is grown at altitudes ranging from 0 to over 1000 m, with the preferred suitable altitude range being up to 750 m, as orchards at higher altitudes produce lower quality hazelnuts [2]. Geographically, the hazelnut is grown almost everywhere in the world, except for the area around the equator. The largest producer worldwide is Turkey, which produces about 62% of the world’s hazelnuts, followed by Italy (13%), the USA (6%), and Azerbaijan (5%) [3]. Hazelnuts are mainly sold to processing companies as shelled nuts, while fresh consumption (in shell) accounts for only 10% of the total harvest. The world’s largest consumer buyer of hazelnuts is Ferrero SpA, which takes about 25% of the global production [2].

The hazelnut is widely used in the bakery, chocolate, and confectionery industries and is also used as an ingredient in many other processed foods [4]. Its consumption is associated with several human health benefits due to the high concentration of bioactive compounds, including sterols, tocopherols, and phenolic compounds [5,6,7,8].

Phenolic compounds are secondary metabolites and important determinants of sensory and additional nutritional quality and health benefits of fruits, vegetables, and other plants. They contribute to the color, flavor, odor, bitterness, astringency, and oxidative stability of foods [9]. Phenolic compounds consist of an aromatic ring bearing one or more hydroxyl groups. Their structures can range from a simple phenolic molecule with one phenolic ring to a complex high molecular mass polymer with multiple hydroxyl groups on aromatic rings [10]. Phenolic compounds have been categorized based on their basic skeleton as follows: simple phenols and benzoquinones (C6); phenolic acids (C6-C1); acetophenones and phenylacetic acids (C6-C2); hydroxycinnamic acids, coumarins, phenylpropanes, and chromones (C6-C3); naphthoquinones (C6-C4); xanthones (C6-C1-C6); stilbenes and anthraquinones (C6-C2-C6); flavonoids and isoflavonoids (C6-C3-C6); catechol melanins ((C6)^n^); lignans and neolignans ((C6-C3)^2^); lignins ((C6-C3)^n^); bioflavonoids ((C6-C3-C6)^2^); and condensed tannins ((C6-C3-C6)^n^) [11]. The main groups of phenolic compounds are phenolic acids, flavonoids, tannins, stilbenes, and lignans. Phenolic acids are further divided into hydroxybenzoic acids and hydroxycinnamic acids [12]. The basic structure of flavonoids is the flavan nucleus, which consists of 15 carbon atoms arranged in three rings. Based on different patterns of methylation, hydroxylation, and conjugation with various mono- and disaccharides, flavonoids are divided into several subclasses, including flavonols, flavones, flavanones, isoflavones, flavanols or flavan-3-ols, and anthocyanidins [10]. The higher the total phenolic content, the higher the observed antioxidant activity [13].

Polyphenols play important roles in human health and disease prevention, so eating fruits, vegetables, and cereals rich in dietary phenols can help reduce the risk of disease and promote human health. The hazelnut is listed as one of the richest sources of polyphenols, along with various plant species, dried herbs, cocoa products, dark-colored berries, seeds, and vegetables [3,9]. Hazelnuts were found to be rich in flavonoids, especially procyanidin dimers and trimers, catechin, epicatechin, and epicatechin-3-gallate [4]. Apart from flavonoids, hazelnuts also contain hydrolysable tannins (glansreginin A and glansreginin B) and phenolic acids (protocatechuic, gallic, *p*-coumaric, and ferulic acids) [8,14]. Beside phenolic compounds, hazelnut kernels contain many other quality nutrients such as unsaturated fatty acids, minerals, vitamins, and fiber. This mean they have a lot of beneficial health effects, which have been proven by numerous clinical studies that have demonstrated the benefits of such bioactive compounds for human health [15,16,17]. Phenolic compounds have the potential to protect against some degenerative diseases such as cancers and diabetes, as well as against cardiovascular diseases [18,19]. They also act as antiallergens, antimicrobials, anti-inflammatories, and antioxidants, among others. Phenolic compounds are also effectively used as functional ingredients in foods, for the prevention of bacterial growth and mold, and for lipid oxidation [20,21].

As a result, the demand for hazelnuts is increasing every year, especially in the bakery industry. At the same time, efforts are currently underway to individuate new productive areas.

It is well known that different climatic conditions affect the synthesis of primary and secondary metabolites [22,23,24]. Increased phenolic synthesis can be due to exposure to extreme temperatures, UV radiation, infection by parasites and pathogens, wounding, and air pollution [25,26,27].

The aim of this study was to determine the variability of the phenolic contents in the kernels of 5 commercial hazelnut cultivars, depending on their growing conditions and location.

## 2. Results

### 2.1. Identification of Phenolic Compounds

There were 13 phenolic compounds identified in the hazelnut kernels, including 7 flavanols, 2 hydroxybenzoic acids, 3 flavonols, and 1 dihydrochalcone. The identified compounds, along with their fragment ions and absorbance spectra, are presented in Table 1.

### 2.2. Phenolic Compounds in Hazelnut Kernels

#### 2.2.1. Contents of Individual Phenolic Compounds

A comparison of the contents of individual phenolic compounds in the kernels of different cultivars grown in six different regions of Europe is shown in Table 2.

Among the determined phenolic compounds, catechin showed the highest content values in most cultivar production area combinations, ranging from 11.66 to 67.87 mg/kg FW of the kernels.

The highest content of catechin was found in the cultivar ‘Pauetet’ from northern Italy, followed by ‘Barcelona’ from Spain and ‘Tonda di Giffoni’ from northern Italy. On the opposite side, there were the cultivars ‘Tonda di Giffoni’ from France, ‘Merveille de Bollwiller’ from northern Italy, and ‘Tonda di Giffoni’ from Slovenia, which had the lowest catechin contents in the kernels.

At all locations, catechin was the major phenolic compound for the cultivars ‘Barcelona’, ‘Pauetet’, and ‘Tonda Gentile delle Langhe’. Similarly, catechin was found as the most abundant phenolic compound in the kernels of ‘Merveille de Bollwiller’ from France, Slovenia, and Spain. In the same cultivar grown in northern Italy, the major phenolic compound was gallic acid, while the Portuguese ‘Merveille de Bollwiller’ contained the greatest amount of procyanidin dimer 2. In the cultivar ‘Tonda di Giffoni’, catechin was the major phenolic compound in the kernels from both locations in Italy, Spain, and Portugal, while protocatechuic acid was most abundant in the kernels from France and gallic acid appeared in the highest concentrations in ‘Tonda di Giffoni’ from Slovenia.

#### 2.2.2. Contents of Different Phenolic Groups

**Figure 1:**
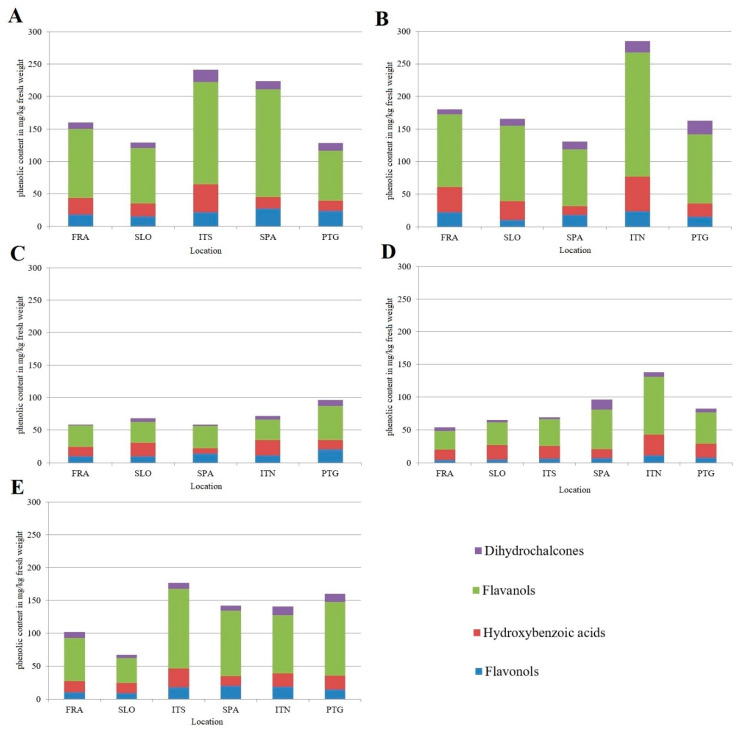
A comparison of the contents of different phenolic groups in the kernels of five different cultivars, (**A**) ‘Barcelona’, (**B**) ‘Pauetet’, (**C**) ‘Merveille de Bollwiller’, (**D**) ‘Tonda di Giffoni’, and (**E**) ‘Tonda gentile delle Langhe’, grown in six different regions of Europe. FRA, France; ITS, central Italy; ITN, north Italy, SLO, Slovenia; SPA, Spain; PTG, Portugal.

As we can see in Figure 1, the flavanols were the major phenolic groups in all hazelnut kernels from all studied sites, as they accounted for more than 50% of all identified phenolic compounds. The flavanols were followed by hydroxybenzoic acids, flavonols, and dihydrochalcones. Considering the individual cultivars, the highest concentrations of phenolics were found in ‘Pauetet’ and ‘Barcelona’, followed by ‘Tonda Gentile delle Langhe’ and ‘Tonda di Giffoni’, while the lowest content was determined in ‘Merveille de Bollwiller’, which also showed the lowest variability with respect to the growing location. The kernels of ‘Pauetet’ cultivated in northern Italy had the highest sum of phenols. This cultivar was followed by ‘Barcelona’ plots from central Italy and Spain. Looking at the individual phenolic groups, the highest contents of all individual phenolic groups studied were found in ‘Pauetet’ and ‘Barcelona’, with the location affecting only the phenolic group composition and not the total phenolic content. The highest contents of flavonols were found in cultivars grown in Spain and Portugal, while the cultivars from northern and central Italy showed the highest contents of hydroxybenzoic acids. The lowest contents of total phenolic compounds as well as flavonols were determined in cultivars grown in Slovenia.

#### 2.2.3. Principal Component Analysis Overview and Relationships between the Variables and Working Hypotheses

**Figure 2:**
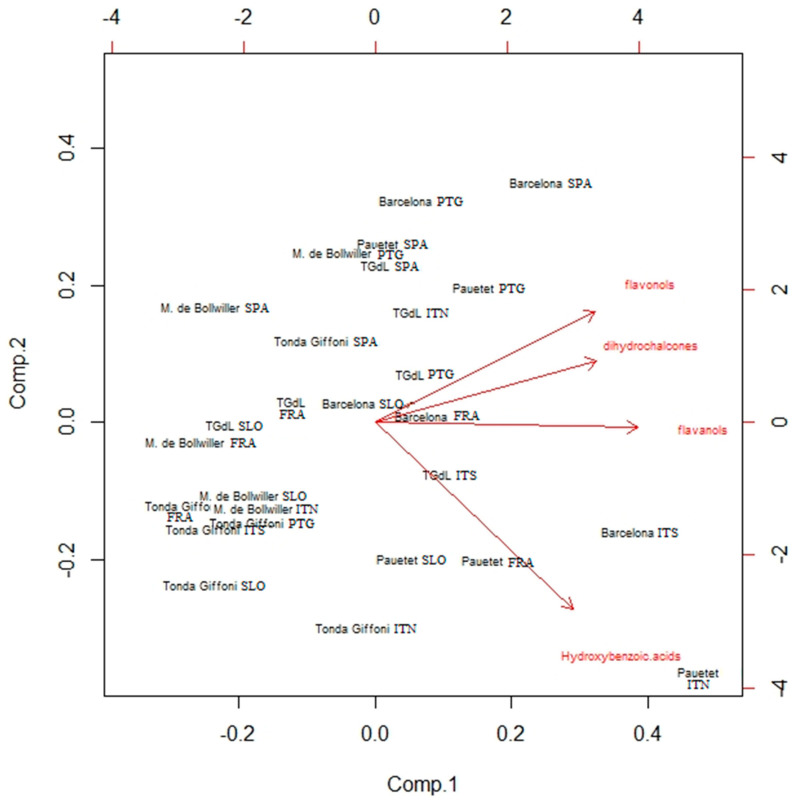
The PCA analysis of the content of four phenolic groups identified in different cultivars from six different regions of Europe.

The PCA, performed for five cultivars that had significantly different contents of phenolic compounds, depending on the growing sites, showed two components, explaining 85.3% of the total variation. In the PCA plot (Figure 2), the cultivar ‘Pauetet’ from all studied locations and ‘Barcelona’ from all locations except Slovenia, are in the 2nd and 4th quadrants, confirming the high contents of all four phenolic groups. On the other hand, the cultivars ‘Merveille de Bollwiller’ and ‘Tonda di Giffoni’ from all growing sites are located in the 1st and 3rd quadrants of the PCA plot, which confirms that the lowest phenolic contents are for these cultivars. The influence of the growing site is also strongly evident in the cultivars that grew in Slovenia. Here, all but ‘Pauetet’ are located in the 1st and 3rd quadrants of the PCA plot, showing the lowest content of phenolic compounds.

#### 2.2.4. Effects of Environmental Factors on Phenolic Contents

Since only two cultivars (‘Tonda di Giffoni’ and ‘Tonda Gentile delle Langhe’) were grown in the sites we considered, the correlations between the variables defining the climatic data for each growing site and the phenolic content were assessed for these two cultivars (Table 3 and Table 4).

Apart from the obvious assumptions that higher monthly solar irradiation determines higher annual solar irradiation, and the fact that the soil pH is lower and negatively affected by rainfall, a correlation between the sums of the phenolic and flavanol contents was observed for both cultivars, while the correlation between the sums of the phenolic and flavanol contents was observed for ‘Tonda di Giffoni’. Interestingly, a strong negative correlation was observed between the hydroxybenzoic acid content and the number of plants per hectare for ‘Tonda di Giffoni’ but not for ‘Tonda Gentile delle Langhe’, while there was a positive correlation between the hydroxybenzoic acid content and altitude for ‘Tonda Gentile delle Langhe’ but not for ‘Tonda di Giffoni’. There was a positive correlation between the dihydrochalcone content and the soil pH in ‘Tonda di Giffoni’ and a negative correlation between the dihydrochalcone content and the annual rainfall. This was not observed for ‘Tonda Gentile delle Langhe’, but there was a positive correlation between the contents of flavanols and flavonols, the total phenolic content, and the solar irradiation per year and per month.

## 3. Discussion

### 3.1. Identification of Phenolic Compounds

Seven flavanols were identified in hazelnut kernels. Here, (+)-catechin and (−)-epicatechin were identified by comparing the retention times with the standard and fragmentation patterns from *m/z* 289 to *m/z* 245. Two *m/z* 577 (M-H)^−^ procyanidin dimers and three *m/z* 865 (M-H)^−^ procyanidin trimers were also detected in the kernel samples. Both hydroxybenzoic acids were confirmed using a commercially available standard. The protocatechuic acid showed a negative parent ion *m/z* 153 and its fragmentation resulted in product ion *m/z* 109, while the gallic acid gave a molecular ion at *m/z* 169 and a fragment ion of *m/z* 125, consistent with gallic acid. For quercetin pentoside, the parent ion at *m/z* 433 resulted in the loss of a pentose group (−132 Da), while quercetin-3-rhamnose showed a negative parent ion *m/z* 447, resulting in a loss of the rhamnose moiety (−146 Da). In both cases, the aglycone was identified as quercetin, as it gave the MS^3^ fragment ions at *m/z* 179, 151, and 121, as observed with the fragmentation of the standard. The negative ESI mass spectrum displayed an ion at *m/z* 463, which produced a fragment ion at *m/z* 317 in the MS^2^ spectrum. The loss at 146 Da indicated the presence of a rhamnose residue associated with the aglycone. To confirm that it was indeed myricetin, the ion was further fragmented at *m/z* 317. The fragmentation yielded ions at *m/z* 289, 179, 151, and 137, confirmed that it was a myricetin aglycone, confirming that the identified compound was myricetin-3-rhamnoside. The single dihydrochalcone was identified using a commercially available standard that gave the same absorbance spectra and fragment ions as the identified compound.

Protocatechuic and gallic acids have already been found in hazelnut kernels. Gallic acid was reported as the most abundant phenolic acid in hazelnut skins, contributing 95% of the total polyphenols in six table cultivars [3]. Moreover, the presence of catechin and dihydrochalcone phloridzin, as well as the flavonols quercetin penthoside, quercetine-3-rhamnoside, and myricetin-3-O-rhamnoside, was described by Slatnar et al. [14].

### 3.2. Phenolic Compounds in Hazelnut Kernels

As previously reported by Jakopic et al. [28], Slatnar et al. [14], and Solar and Stampar [29], catechin and procyanidin dimers were the major phenolic compounds found in hazelnut kernels, which is in agreement with our results. The highest contents of catechin in all cultivars and locations studied here were determined in Spain and in northern Italy, and the lowest were in Slovenia and France. This corresponded to the results for the contents of phenolic groups, where the flavanol contents in the cultivars from Spain and northern Italy were higher compared to the cultivars from France and Slovenia. This could be due to the fact that the solar irradiation values are higher at northern latitudes (Table 5), which probably has a positive effect on the flavonoid contents in plants, as already reported by Jakopic et al. [30] and Jaakola and Hohtola [31] and also shown in the correlation table for the ‘Tonda Gentile delle Langhe’ cultivar (Table 3). This can be explained by the fact that phenols are ultraviolet-absorbing compounds synthesized in response to higher solar radiation to protect plant cells from excessive UV-B radiation [26,32]. The highest contents of flavonols being observed in cultivars grown in Spain and Portugal can be explained by these locations having the strongest solar irradiation levels, as already been described for the flavanol contents. The highest contents of hydroxybenzoic acids among all cultivars studied were found in the cultivars from northern and central Italy. As can be seen from Table 5, they grew at the highest altitudes. According to the observations made by Senica et al. [33] that high-altitude conditions stimulate the synthesis of phenolic compounds, from this we can assume that the high contents of hydroxybenzoic acids in the hazelnut kernels from both Italian collection orchards were a result of growing at higher altitudes. Such a conclusion can also be confirmed by the correlation table for ‘Tonda Gentile delle Langhe’, which shows that the altitude positively affected the content of hydroxybenzoic acids (Table 3). The same linkage can be seen in the cultivars from Spain, which grew at a low altitude and had low contents of hydroxybenzoic acids. The lowest content of phenolic compounds was found in Slovenia, which can be easily explained by the fact that the orchard is located at a lower altitude, resulting in a lower content of phenolic acids, as explained earlier. The low phenolic content in Slovenia can also be attributed to the fact that the orchard site receives more rainfall than other growing areas and less solar irradiation, both of which have a negative effect on the synthesis of phenolic compounds [26,32,33]. As in our study, a negative correlation was found between the dihydrochalcone content and annual rainfall by Bars-Cortina et al. [34], which was thought to be due to hydric stress. A positive strong correlation between the soil pH and dihydrochalcone content has not been noticed or excluded in other studies so far, and we believe it should be further investigated, as our results showed that higher pH values could stimulate dihydrochalcone synthesis.

## 4. Materials and Methods

### 4.1. Plant Material

**Figure 3:**
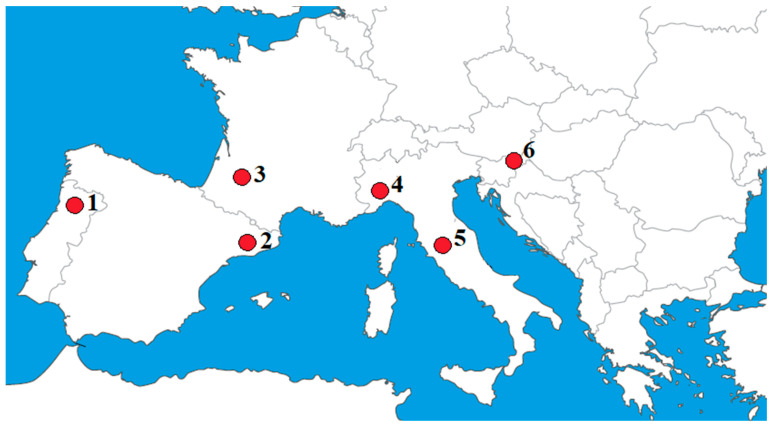
Locations of hazelnut sampling for the phenolic analysis: 1—Vila Real, Portugal (PTG); 2—Constanti, Spain (SPA); 3—Puéchoursi and Montesqieu, France (FRA); 4—Cravanzana, Italy (ITN); 5—Viterbo, Italy (ITS); 6—Maribor, Slovenia (SLO).

Five cultivars, namely ‘Tonda Gentile delle Langhe’, ‘Merveille de Bollwiller’, ‘Pauetet’, ‘Tonda di Giffoni’, and ‘Barcelona’ (syn. ‘Fertile de Coutard’, ‘Castanyera’, ‘Grada’) were included in the study. Kernel samples were collected in five European countries, namely Italy, France, Spain, Portugal, and Slovenia. In Italy, the ENEA (ITS) and University of Torino (ITN) collected the samples in Le Cese (Viterbo) and Cravanzana (Cuneo), respectively. In France (FRA), the nuts were provided by the ANPN from Puéchoursi and Montesqieu collections. In Spain (SPA), the samples were taken by the IRTA Mas Bove in Constantí germplasm collection. In Portugal (PTG), the UTAD provided samples from the collection in Vila Real, and in Slovenia (SLO), the Biotechnical Faculty took samples at the Maribor collection orchard (Figure 3). All samples were collected in the same growing season. The locations differ in their geographical and climatological features, soil properties, and orchard characteristics, which are detailed in Table 5.

The plants were maintained in a randomized block design with three replicates for each cultivar. The nuts were harvested at maturity in early September, and a sample of about 1 kg was randomly chosen for each cultivar at each growing site. The nuts were dried according to a standard procedure to 12% moisture and stored for four months in a cold room at approximately 10 °C. The cracking was achieved by hand. Four repetitions for each accession were done. Twenty raw kernels (kernel + pellicle) for each repetition were randomly selected for further analysis of the phenolic compounds.

### 4.2. Chemicals

For the phenolic compound quantification the following standards were used: procyanidin B2 and myricetin-3-rhamnoside from Sigma (St. Louis, MO, USA); quercitrin (quercetin-3-*O*-rhamnoside), quercetin-3-*O*-glucoside, phloridzin dihydrate, and (−)-epicatechin from Fluka Chemie GmBH (Buchs, Switzerland); gallic acid and protocatechuic acid from Merck (Darmstadt, Germany); and (+)-catechin from Roth (Karlsruhe, Germany). The methanol used for the extraction of phenolic compounds and *n*-hexane for oil removal were acquired from Sigma. The chemicals used for the mobile phases were HPLC-MS-grade acetonitrile and formic acid from Fluka Chemie GmbH. The water used for the mobile phase was bidistilled and purified with the Milli-Q system (Millipore, Bedford, MA, USA).

### 4.3. Extraction of Phenolic Compounds from Hazelnut Kernels

The extraction was performed as described by Mikulic-Petkovsek et al. [35], with minor modifications. The hazelnut samples were ground with a mechanical grinder. Here, 5 g of the hazelnut crumbs was extracted for 60 min with 15 mL of methanol containing 1% 2,6-di-tert-butyl-4-methylphenol (BHT) in an ice-cooled water bath using sonification. The hazelnut extracts were centrifuged (Eppendorf centrifuge 5810 R, Hamburg, Germany) at 10,000 rpm for 10 min at 4 °C and the supernatant was filtered through a 0.45 μm membrane filter (Macherey-Nagel, Düren, Germany). An additional technique was performed according to Pirisi et al. [36] and Chan and Ismail [37] with some modifications. The supernatant was mixed with 10 mL of *n*-hexane for 5 min in a vortex machine. The mixture was transferred to a separatory funnel where the methanol and hexane layer were separated. The procedure was repeated twice with 10 mL of *n*-hexane. The methanol extract was concentrated in a rotary evaporator (Büchi Rotavapor R-114 and Büchi Vacobox B-171; Flawil, Switzerland) under reduced pressure at 337 mbar. The dry residue was then dissolved in 1.5 mL of methanol.

The phenolic compounds were analyzed on a Thermo Finnigan Surveyor HPLC system (San Jose, CA, USA) with a diode array detector at 280 and 350 nm. The hydroxybenzoic acids (gallic acid, protocatechuic acid), dihydrochalcone (phloridzin), and flavanols (catechin, epicatechin, all procyanidins) were detected at 280 nm, whereas the quercetin pentoside, quercetin-3-rhamnoside (quercitrin), and myricetin-3-rhamnoside were estimated at 350 nm. The spectra of the compounds were recorded between 200 and 500 nm. The compounds were separated on a Phenomenex Gemini C_18_ (150 mm × 4.60 mm, 3 micron) (Torrance, CA, USA) column operated at 25 °C with elution solvents A (1% formic acid in water) and B (100% acetonitrile), and the flow-rate was 1 mL/min. The following gradient method was used: 0–5 min, 3–9% B; 5–15 min, 9–16% B, 15–45 min, 16–50% B; 45–50 min, 50% isocratic; and finally washing and reconditioning of the column [38]. The injection amount of extract was 20 μL. The identification of compounds was achieved by comparing retention times and spectra, as well as via the addition of an internal standard. The phenolic compounds were confirmed using the Thermo Scientific LCQ Deca XP mass spectrometer with an electrospray interface (ESI) operating in negative ion mode. The concentrations of phenolic compounds were calculated from the peak areas of the samples and the corresponding standards. An unknown procyanidin dimer was quantified and expressed in procyanidin B2 equivalents. The quercetine penthoside was quantified and expressed as quercetine 3-*O*-rhamnoside. The concentrations were expressed in mg per kg of hazelnut kernels.

## 5. Conclusions

Catechin and procyanidin dimers were the major phenolic compounds determined in hazelnut kernels. The highest contents of catechin and overall flavanols in all cultivars and locations studied were found in Spain and northern Italy, and the lowest in Slovenia and France. Higher contents of flavanols and flavonols corresponded to growing areas with higher solar irradiation, which increased their content levels. The content of hydroxybenzoic acids correlated with altitude, which stimulated phenolic acid synthesis. A negative correlation was observed between the dihydrochalcone content and annual rainfall, probably due to hydric stress, while the observed positive strong correlation between the soil pH and dihydrochalcone content should be further investigated, as higher pH values could stimulate dihydrochalcone synthesis.

## Figures and Tables

**Table 1:** The identification of phenolic compounds in hazelnut kernels in negative ion mode using HPLC-MS.

Tentative Identification	Λ (nm)	[M-H]^−^ (*m/z*)	MS^2^ (*m/z*)
Flavanols			
Catechin	234,279	289	245
Epicatechin	234,279	289	245
Procyanidin dimer 1	235,28	577	425,407,289
Procyanidin dimer 2	234,279	577	425,407,289
Procyanidin trimer 1	234,278	865	577,451,425,407,289
Procyanidin trimer 2	234,278	865	577,451,425,407,289
Procyanidin trimer 3	234,28	865	577,425,407,289
Hydroxybenzoic acids			
Gallic acid	271	169	125
Protocatechuic acid	259,264	153	109
Flavonols			
Quercetin pentoside	256,356	433	301
Quercetin-3-rhamnoside	255,358	447	301
Myricetin-3-rhamnoside	255,349	463	317
Dihydrochalcones			
Phloridzin (Phloretin-2-glucoside)	230,285	435	273

Note: λ, absorbance spectra of the compounds; [M-H]^−^, pseudomolecular ion identified in negative ion mode; MS^2^, fragment ions obtained from pseudomolecular ion in negative ion mode.

**Table 2:** The contents of individual phenolic compounds in hazelnut kernels cultivated in different regions of Europe.

Cultivar	Compound	Phenolic Compounds Content in Relation to the Cultivation Area in mg/kg Fresh Weight					
‘Barcelona’		FRA	ITS	ITN	SLO	SPA	PTG
	Quercetine penthoside	0.38 ± 0.03 b	0.25 ± 0.01 a	nd	0.26 ± 0.02 a	0.36 ± 0.02 b	0.27 ± 0.01 a
	Quercetine rhamnoside	13.14 ± 1.40 a	14.14 ± 1.23 a	nd	12.68 ± 0.36 a	19.51 ± 1.69 b	15.4 ± 0.81 a
	Myricetin-3-rhamnoside	4.68 ± 0.47 b	7.58 ± 0.38 c	nd	2.26 ± 0.75 a	7.66 ± 0.54 c	8.19 ± 0.37 c
	Gallic acid	12.98 ± 1.35 cd	15.47 ± 1.10 d	nd	10.07 ± 0.60 c	3.9 ± 0.95 a	7.68 ± 0.29 b
	Protocatechuic acid	12.08 ± 0.58 a	27.14 ± 4.70 b	nd	10.25 ± 2.75 a	13.62 ± 2.47 a	8.02 ± 2.40 a
	Catechin	29.39 ± 2.66 a	36.66 ± 5.70 a	nd	31.91 ± 2.12 a	63.81 ± 4.55 b	26.19 ± 3.61 a
	Epicatechin	10.96 ± 2.99 ab	13.31 ± 1.89 b	nd	8.56 ± 2.44 a	18.89 ± 1.28 c	8.00 ± 0.49 a
	Procyanidin dimer 1	9.04 ± 0.11 ab	16.42 ± 3.49 c	nd	7.65 ± 0.84 ab	3.92 ± 0.51 a	9.07 ± 2.95 ab
	Procyanidin dimer 2	20.58 ± 2.80 b	33.32 ± 4.68 c	nd	14.85 ± 0.54 a	30.42 ± 0.21 c	9.59 ± 2.61 a
	Procyanidin trimer 1	13.80 ± 0.26 b	14.96 ± 3.00 b	nd	6.54 ± 0.80 a	20.91 ± 1.80 c	9.79 ± 0.44 a
	Procyanidin trimer 2	13.10 ± 0.89 b	19.46 ± 2.87 c	nd	11.63 ± 1.76 ab	11.62 ± 0.95 ab	8.49 ± 3.11 a
	Procyanidin trimer 3	9.71 ± 0.46 a	23.67 ± 2.77 c	nd	3.77 ± 0.33 a	16.65 ± 1.22 b	5.93 ± 1.39 a
	Phloridzin	10.18 ± 1.62 ab	18.99 ± 1.64 c	nd	8.53 ± 2.12 a	12.31 ± 3.29 ab	11.95 ± 0.86 ab
‘Pauetet’							
	Quercetine penthoside	0.83 ± 0.01 c	nd	0.96 ± 0.10 c	0.64 ± 0.05 b	0.32 ± 0.01 a	0.38 ± 0.02 a
	Quercetine rhamnoside	8.25 ± 0.43 a	nd	12.61 ± 1.00 b	7.79 ± 0.10 a	9.46 ± 1.16 a	8.61 ± 0.21 a
	Myricetin-3-rhamnoside	13.53 ± 0.80 d	nd	10.17 ± 0.80 c	2.20 ± 0.08 a	8.37 ± 0.76 bc	6.58 ± 0.30 b
	Gallic acid	15.74 ± 0.94 d	nd	14.85 ± 1.33 cd	8.34 ± 0.75 b	2.54 ± 0.20 a	12.83 ± 0.49 c
	Protocatechuic acid	22.67 ± 1.25 b	nd	38.64 ± 2.15 c	20.39 ± 5.28 b	11.06 ± 1.75 a	7.43 ± 0.43 a
	Catechin	43.21 ± 2.55 b	nd	67.87 ± 5.70 c	40.20 ± 5.65 b	31.82 ± 1.67 a	33.32 ± 2.68 a
	Epicatechin	9.89 ± 0.42 a	nd	15.22 ± 1.87 b	14.10 ± 1.26 b	11.11 ± 0.79 a	14.78 ± 0.66 b
	Procyanidin dimer 1	12.01 ± 0.72 bc	nd	21.28 ± 4.64 d	5.86 ± 0.95 ab	1.99 ± 0.27 a	14.70 ± 0.34 cd
	Procyanidin dimer 2	10.22 ± 0.57 a	nd	23.04 ± 5.03 b	18.57 ± 2.92 ab	16.38 ± 3.35 ab	8.46 ± 1.49 a
	Procyanidin trimer 1	4.04 ± 0.17 a	nd	15.90 ± 1.36 c	14.18 ± 1.76 c	11.38 ± 1.99 b	9.12 ± 1.78 b
	Procyanidin trimer 2	7.43 ± 0.56 a	nd	32.01 ± 5.52 b	12.24 ± 3.13 a	6.91 ± 0.79 a	11.78 ± 0.29 a
	Procyanidin trimer 3	24.63 ± 2.11 c	nd	15.20 ± 0.36 b	10.41 ± 1.79 ab	7.23 ± 1.40 a	13.63 ± 0.60 ab
	Phloridzin	7.99 ± 1.02 a	nd	17.53 ± 1.57 b	10.49 ± 1.89 a	12.14 ± 2.32 a	21.18 ± 3.18 c
‘Merveille de Bollwiller’							
	Quercetine penthoside	0.49 ± 0.04 a	nd	0.35 ± 0.04 a	0.83 ± 0.08 b	0.21 ± 0.01 a	0.77 ± 0.03 b
	Quercetine rhamnoside	6.97 ± 0.50 a	nd	7.35 ± 0.74 ab	6.44 ± 0.57 a	8.92 ± 0.52 b	11.94 ± 0.53 c
	Myricetin-3-rhamnoside	2.71 ± 0.18 a	nd	3.89 ± 0.52 b	2.50 ± 0.38 a	4.69 ± 0.38 b	7.46 ± 0.19 c
	Gallic acid	3.88 ± 0.20 a	nd	16.80 ± 1.65 c	9.32 ± 0.66 b	3.01 ± 0.09 a	4.64 ± 0.12 a
	Protocatechuic acid	10.06 ± 0.54 b	nd	6.10 ± 0.81 a	11.15 ± 0.85 b	5.12 ± 0.49 a	9.91 ± 1.60 b
	Catechin	11.66 ± 1.96 ab	nd	9.75 ± 0.84 a	14.44 ± 1.75 c	13.41 ± 0.53 bc	11.15 ± 0.55 ab
	Epicatechin	1.55 ± 0.04 a	nd	5.52 ± 1.67 bc	1.55 ± 0.07 a	3.40 ± 0.13 ab	6.49 ± 0.43 c
	Procyanidin dimer 1	3.60 ± 0.62 b	nd	1.55 ± 0.42 a	1.93 ± 0.30 a	1.34 ± 0.07 a	7.43 ± 0.14 c
	Procyanidin dimer 2	3.58 ± 0.48 a	nd	3.35 ± 1.52 a	8.94 ± 0.91 b	8.01 ± 0.45 b	15.95 ± 0.46 c
	Procyanidin trimer 1	3.93 ± 0.20 b	nd	4.33 ± 1.22 b	1.33 ± 0.15 a	2.50 ± 0.13 ab	2.81 ± 0.64 ab
	Procyanidin trimer 2	2.16 ± 0.28 a	nd	3.35 ± 0.56 b	1.75 ± 0.21 a	2.52 ± 0.13 ab	4.61 ± 0.08 c
	Procyanidin trimer 3	6.51 ± 0.16 d	nd	3.95 ± 0.33 c	2.62 ± 0.33 a	3.02 ± 0.06 ab	3.63 ± 0.19 bc
	Phloridzin	1.08 ± 0.12 a	nd	5.20 ± 0.75 b	5.56 ± 0.27 b	2.24 ± 0.08 a	9.29 ± 0.42 c
‘Tonda di Giffoni’							
	Quercetine penthoside	0.43 ± 0.01 b	0.37 ± 0.01 ab	0.78 ± 0.10 c	0.34 ± 0.03 ab	0.35 ± 0.02 ab	0.27 ± 0.03 a
	Quercetine rhamnoside	2.52 ± 0.17 a	3.81 ± 0.09 bc	5.71 ± 0.40 d	3.12 ± 0.22 ab	3.92 ± 0.20 c	4.49 ± 0.22 c
	Myricetin-3-rhamnoside	1.06 ± 0.09 a	2.44 ± 0.14 c	4.51 ± 0.28 e	1.71 ± 0.08 b	3.01 ± 0.35 cd	3.18 ± 0.16 d
	Gallic acid	3.93 ± 0.12 a	8.51 ± 0.06 b	21.63 ± 1.80 d	14.96 ± 1.52 c	3.39 ± 0.63 a	7.85 ± 1.88 b
	Protocatechuic acid	11.81 ± 0.24 b	9.80 ± 0.65 a	10.16 ± 1.39 b	6.38 ± 0.43 a	9.87 ± 0.85 b	12.94 ± 1.75 b
	Catechin	8.06 ± 0.05 a	17.48 ± 1.64 b	47.35 ± 2.05 d	10.63 ± 0.60 a	25.42 ± 1.31 c	18.53 ± 1.83 b
	Epicatechin	5.01 ± 0.34 a	4.32 ± 0.99 a	7.93 ± 0.99 b	4.45 ± 0.35 a	5.23 ± 0.83 a	3.77 ± 0.51 a
	Procyanidin dimer 1	3.27 ± 1.14 ab	4.91 ± 0.11 b	5.13 ± 1.19 b	4.31 ± 0.18 b	1.99 ± 0.20 a	4.36 ± 0.21 b
	Procyanidin dimer 2	3.72 ± 0.20 a	4.00 ± 0.17 a	8.08 ± 0.84 bc	4.76 ± 0.60 ab	13.96 ± 2.44 d	9.76 ± 1.15 c
	Procyanidin trimer 1	3.12 ± 0.17 ab	3.97 ± 0.38 ab	5.58 ± 0.83 bc	2.62 ± 0.35 a	5.85 ± 0.60 bc	3.09 ± 0.47 ab
	Procyanidin trimer 2	1.77 ± 0.47 a	2.96 ± 0.48 ab	4.75 ± 0.33 c	4.44 ± 0.18 bc	3.13 ± 0.32 abc	3.97 ± 0.26 bc
	Procyanidin trimer 3	3.52 ± 0.10 a	3.36 ± 0.23 a	9.62 ± 0.56 b	3.78 ± 0.60 a	4.54 ± 0.76 a	4.45 ± 0.31 a
	Phloridzin	5.39 ± 2.46 a	3.12 ± 0.99 a	6.72 ± 2.34 a	3.43 ± 0.35 a	15.88 ± 1.49 b	5.71 ± 1.74 a
‘Tonda gentile delle Langhe’							
	Quercetine penthoside	0.33 ± 0.01 ab	0.54 ± 0.06 d	0.45 ± 0.03 cd	0.27 ± 0.02 a	0.40 ± 0.02 bc	0.41 ± 0.02 bc
	Quercetine rhamnoside	5.81 ± 0.19 a	9.82 ± 0.82 c	9.35 ± 0.52 bc	4.62 ± 0.10 a	8.71 ± 0.85 bc	7.69 ± 0.20 b
	Myricetin-3-rhamnoside	4.48 ± 0.12 a	7.87 ± 0.58 bc	9.02 ± 0.26 c	4.48 ± 0.38 a	11.30 ± 1.19 d	6.78 ± 0.13 b
	Gallic acid	8.01 ± 1.48 b	15.83 ± 0.62 d	10.67 ± 0.41 c	9.76 ± 0.22 c	3.35 ± 0.10 a	9.75 ± 1.38 bc
	Protocatechuic acid	8.31 ± 0.57 b	12.50 ± 1.32 c	9.19 ± 0.59 b	5.04 ± 0.47 a	11.15 ± 1.79 bc	10.69 ± 0.03 bc
	Catechin	21.48 ± 2.70 a	35.44 ± 3.73 bc	30.00 ± 4.27 ab	18.16 ± 1.34 a	37.52 ± 6.22 bc	43.97 ± 5.17 c
	Epicatechin	5.34 ± 1.66 a	16.19 ± 2.05 c	16.21 ± 1.07 c	1.40 ± 0.28 a	15.97 ± 1.37 c	11.00 ± 2.65 b
	Procyanidin dimer 1	9.52 ± 0.70 b	11.36 ± 2.14 b	8.87 ± 1.24 b	3.21 ± 0.32 a	2.69 ± 0.27 a	10.55 ± 2.70 b
	Procyanidin dimer 2	9.26 ± 1.98 bc	17.88 ± 1.29 de	8.64 ± 1.83 b	3.70 ± 0.33 a	15.83 ± 1.56 d	13.61 ± 1.65 cd
	Procyanidin trimer 1	6.98 ± 2.24 b	10.90 ± 0.94 bc	7.62 ± 0.96 b	1.58 ± 0.50 a	8.02 ± 1.88 b	13.61 ± 1.56 c
	Procyanidin trimer 2	7.36 ± 1.27 b	17.51 ± 1.04 c	8.27 ± 0.98 b	3.30 ± 0.39 a	9.74 ± 1.15 b	9.07 ± 0.90 b
	Procyanidin trimer 3	6.32 ± 1.41 a	12.20 ± 1.97 b	9.45 ± 0.58 ab	6.67 ± 0.11 a	9.87 ± 1.29 ab	10.78 ± 2.21 ab
	Phloridzin	8.94 ± 0.24 b	8.73 ± 0.86 b	12.94 ± 1.29 c	5.41 ± 0.76 a	7.81 ± 1.40 ab	12.59 ± 0.75 c

Data are means ± the standard error. Means followed by different letters within rows are significantly different (*p* < 0.05). Note: nd, no data; FRA, France; ITS, south Italy; ITN, north Italy, SLO, Slovenia; SPA, Spain; PTG, Portugal.

**Table 3:** Correlation table of the variables for the cultivar ‘Tonda di Giffoni’.

‘Tonda di Giffoni’	Plants Per Ha	Soil pH	Rainfall/Year	Solar Irrad./Year	Solar Irrad./Month	Abs. Min Winter T	Abs. Max Summer T	Mean Annual T	Elevation	All Phenolics	Dihydrochalchones	Flavanols	Hydroxybenzoic Acids	Flavonols
Flavonols	−0.514	−0.045	0.107	0.385	0.386	0.096	0.191	−0.102	0.658	0.952 *	0.228	0.950 *	0.729	-
Hydroxybenz. acids	−0.900 *	−0.507	0.492	−0.329	−0.328	−0.205	0.334	−0.733	0.668	0.697	−0.364	0.673	-	
Flavanols	−0.358	0.195	−0.124	0.335	0.336	0.010	0.134	−0.083	0.435	0.998 *	0.402	-		
Dihydrochalchones	0.701	0.909 *	−0.827 *	0.659	0.658	0.187	−0.188	0.653	−0.457	0.384	-			
All phenolics	−0.380	0.170	−0.104	0.303	0.304	0.000	0.153	−0.122	0.436	-				
Elevation	−0.813 *	−0.682	0.754	0.115	0.117	0.302	0.015	−0.289	-					
Mean annual T	0.759	0.657	0.612	0.865 *	0.864 *	0.235	−0.167	-						
Abs. Max Summer T	−0.281	−0.145	−0.113	−0.060	−0.061	−0.829 *	-							
Abs. Min Winter T	0.115	−0.065	0.308	0.338	0.338	-								
Solar irrad./month	0.415	0.486	−0.416	1.000 *	-									
Solar irrad./year	0.416	0.486	−0.416	-										
Rainfall/year	−0.791	−0.960 *	-											
Soil pH	0.815 *	-												
Plants per ha	-													

Note: *, positive correlation betwen variables (*p* < 0.01); -, correlation betwen the same variables (1.000).

**Table 4:** Correlation table of the variables for the cultivar ‘Tonda gentile delle Langhe’.

‘Tonda Gentile Delle Langhe’	Plants Per ha	Soil pH	Rainfall/Year	Solar irrad./Year	Solar irrad./Month	Abs. Min Winter T	Abs. Max Summer T	Mean Annual T	Elevation	All Phenolics	Dihydrochalchones	Flavanols	Hydroxybenzoic Acids	Flavonols
Flavonols	0.078	0.555	−0.179	0.832 *	0.833 *	0.322	−0.202	0.506	0.197	0.791	0.407	0.773	0.373	-
Hydroxybenz. acids	−0.471	−0.459	0.557	0.273	0.275	0.195	−0.045	0.089	0.838 *	0.740	0.354	0.699	-	
Flavanols	0.005	0.144	0.040	0.865 *	0.866 *	0.261	0.052	0.617	0.460	0.994 *	0.558	-		
Dihydrochalchones	−0.482	−0.162	0.062	0.369	0.369	−0.444	0.750	−0.007	0.626	0.597	-			
All phenolics	−0.083	0.104	0.087	0.828 *	0.829 *	0.232	0.066	0.547	0.533	-				
Elevation	−0849 *	−0.685	0.696	−0.010	−0.008	−0.111	0.299	−0.353	-					
Mean annual T	0.780	0.653	−0.622	0.804	0.804	0.199	−0.121	-						
Abs. Max Summer T	−0.284	−0.280	−0.173	−0.087	−0.087	−0.848 *	-							
Abs. Min Winter T	0.174	0.156	0.369	0.370	0.370	-								
Solar irrad./month	0.388	0.568	−0.305	1.000 *	-									
Solar irrad./year	0.389	0.569	−0.306	-										
Rainfall/year	−0.780	−0.812 *	-											
Soil pH	0.757	-												
Plants per ha	-													

Note: *, positive correlation betwen variables (*p* < 0.01); -, correlation betwen the same variables (1.000).

**Table 5:** The geographical, climatic, soil, and orchard characteristics of the six cultivation areas.

Geographical Data				Climatic Data						Soil Traits		Orchard Characteristics			
Country	Town	Latitude Longitude	Altitude (m S.A.l.)	Mean Annual T (°C)	Absolute Summer T (°C)	Absolute Winter T (°C)	Global Solar Irradiation		Rainfall (mm/Year)	pH	Texture	Plants/ha	Training SYSTEM	Irigation	Pest Management
							kWh/m^2^/Month	kWh/m^2^/Year							
France (FRA 1)	Puéchoursi (81470)	N 43°51′16″ E 1.9°10′52″	278	13.8	40.7	−19.2	137.9	1655.0	638	slightly basic	silty clay to silty clayey-sandy	666	Single trunk	Yes	CON
France (FRA 2)	Montesqieu	N 44°21′50″ E 0.4°44′41″	38	13.4	41.0	−21.9	139.8	1677.9	712	basic	silt-clayey-sandy	666	Single trunk	Yes	CON
Italy (ITS)	Viterbo	N 42°20′52″ E 12°11′40″	567	14.1	38.0	−5.1	153.6	1842.8	1073	acid 6.1	sandy clay loam	500	Bush	Yes	IP
Italy (ITN)	Cravanzana	N 44°57′72″ E 8°13′39″	545	11.5	34.1	−13.3	145.5	1745.7	922	slightly acid 6.4	silt loam	333	Bush	No	IP
Slovenia (SLO)	Maribor	N 46°53′94″ E 15°64′50″	275	10.8	33.1	−20.1	128.4	1541.1	1.078	acid 6.0	loam to silty loam	500	Bush	No	IP
Spain (SPA)	Constant	N 41°10′9″ E 1°20′28″	110	15.8	36.2	−6.5	165.1	1981.6	583	>8 alcaline	Loam sandy	833	Single trunk/Bush	Yes	CON
Portugal (PTG)	Vila Real	N 41°9′ E 8°23′	470	13.6	39.8	−6.5	156.9	1883.3	1.000	sub-acid	Medium to Coarse	500	Bush	No	CON

## Data Availability

The data presented in this study are available on request from the corresponding author. The remaining data are not publicly available due to privacy.
